# Hyperspectral data as a biodiversity screening tool can differentiate among diverse Neotropical fishes

**DOI:** 10.1038/s41598-021-95713-0

**Published:** 2021-08-09

**Authors:** M. A. Kolmann, M. Kalacska, O. Lucanus, L. Sousa, D. Wainwright, J. P. Arroyo-Mora, M. C. Andrade

**Affiliations:** 1 University of Michigan, Museum of Paleontology, 1105 N University Ave, Ann Arbor, MI 48109 USA; 2 Royal Ontario Museum, 100 Queens Park, Toronto, ON M5S 2C6 Canada; 3 Applied Remote Sensing Lab, McGill University, Montreal, QC H3A 0B9 Canada; 4 Below Water Inc., Vaudreuil-Dorion, QC J7V 0K4 Canada; 5 Laboratório de Ictiologia de Altamira, Universidade Federal do Pará, Altamira, PA Brazil; 6 Yale University (Peabody Museum), New Haven, CT, USA; 7 National Research Council Canada, Ottawa, ON Canada; 8 Núcleo de Ecologia Aquática e Pesca da Amazônia, Universidade Federal do Pará, Belém, PA Brazil

**Keywords:** Ichthyology, Biodiversity

## Abstract

Hyperspectral data encode information from electromagnetic radiation (i.e., color) of any object in the form of a spectral signature; these data can then be used to distinguish among materials or even map whole landscapes. Although hyperspectral data have been mostly used to study landscape ecology, floral diversity and many other applications in the natural sciences, we propose that spectral signatures can be used for rapid assessment of faunal biodiversity, akin to DNA barcoding and metabarcoding. We demonstrate that spectral signatures of individual, live fish specimens can accurately capture species and clade-level differences in fish coloration, specifically among piranhas and pacus (Family Serrasalmidae), fishes with a long history of taxonomic confusion. We analyzed 47 serrasalmid species and could distinguish spectra among different species and clades, with the method sensitive enough to document changes in fish coloration over ontogeny. Herbivorous pacu spectra were more like one another than they were to piranhas; however, our method also documented interspecific variation in pacus that corresponds to cryptic lineages. While spectra do not serve as an alternative to the collection of curated specimens, hyperspectral data of fishes in the field should help clarify which specimens might be unique or undescribed, complementing existing molecular and morphological techniques.

## Introduction

The biological sciences face two dire and disturbing issues: (1) increases in extinctions of animals across all continents and oceans^1–4^, as well as (2) a shrinking pool of taxonomists trained to identify these organisms^5–7^. The need to find, categorize, and curate biodiversity is both critical for the prioritized deployment of conservation resources and for maintaining a record of biodiversity before such organisms are lost. Complementing surveys and natural history collections, there are methods like DNA barcoding and meta-barcoding which have been developed to catalog biodiversity, for both previously known and unknown organisms^7^. Rapid identification of phenotypic diversity in the field gives biodiversity researchers the ability to streamline their collections and surveys, as well as brings assessment of phenotypic variation back to the forefront of systematics. However, both traditional morphological taxonomy and molecular barcoding still require considerable benchwork: i.e., an intensively trained taxonomist who completes microscopy and meristics to identify phenotypic structures or a technician that performs PCR reactions and sequencing for barcoding. What if another method for rapid identification of polymorphism in natural populations existed? Moreover, what if assessment of phenotypic polymorphism could be undertaken in the field while living organisms are held in person, rather than in a lab far away? We propose that hyperspectral assessment of live organisms, and the generation of spectral signatures for individual taxa (or operational taxonomic units, OTUs) can provide: (1) rapid assessment of phenotypic variability in the field, (2) discriminate organisms along meaningful categories like taxonomy or ontogeny, and (3) complement or even reconcile morphological taxonomy with molecular barcoding methods.

Hyperspectral analysis is a well-established methodology in the Earth sciences with roots in chemistry and physics. By analyzing how light (electromagnetic radiation) interacts with matter (even an organism), it is possible to determine an object’s chemical composition and physical structure. As such, hyperspectral analysis differs from conventional analysis of photographs or other digital media (e.g.^8,9^). Matter reflects, absorbs, and emits radiation in varying proportions at different wavelengths, creating what is known as a *spectral signature*, measured light encoded with information. It is therefore possible to assess the chemical composition of an organism’s anatomy and use this same anatomical region as a point of comparison among other related species. Moreover, whereas existing biodiversity survey methods like DNA barcoding and traditional morphological taxonomy incur sustained costs, like the purchase of reagents, field spectroradiometers represent a one-time cost comparable even in the short-term to molecular labs.

Early applications for biodiversity assessment with spectroradiometry date back six decades to investigations on plants^10^ and reptiles^11^. Since then, it has become a widely applied approach for mapping vegetation biodiversity (e.g.^12^) with large-scale mapping campaigns carried out in a range of ecosystems worldwide (e.g.^13,14^). For studying animals, its application is far less common. Early work examined the potential for hyperspectral analysis to rapidly assess community numbers in remote regions by testing it on polar mammals^15^. Hyperspectral analysis has also been used in a limited number of studies to differentiate amphibian and reptile species^16–18^ and insects^19^. In aquatic environments, this technology is even more rarely used, but early attempts at monitoring coral beds^20–22^, identifying deep sea megafauna^23^ and studying camouflage in marine taxa^24,25^ have shown promising results. Like DNA barcoding, taxonomic spectroradiometry (i.e., spectral signatures) could ‘invert’ traditional taxonomy when used as a ‘taxonomic screening tool’ whereby predefined, spectrally distinctive groups are examined for trait variation according to what phenotypic characters morpho-taxonomists are interested in^26^.

**Figure 1:**
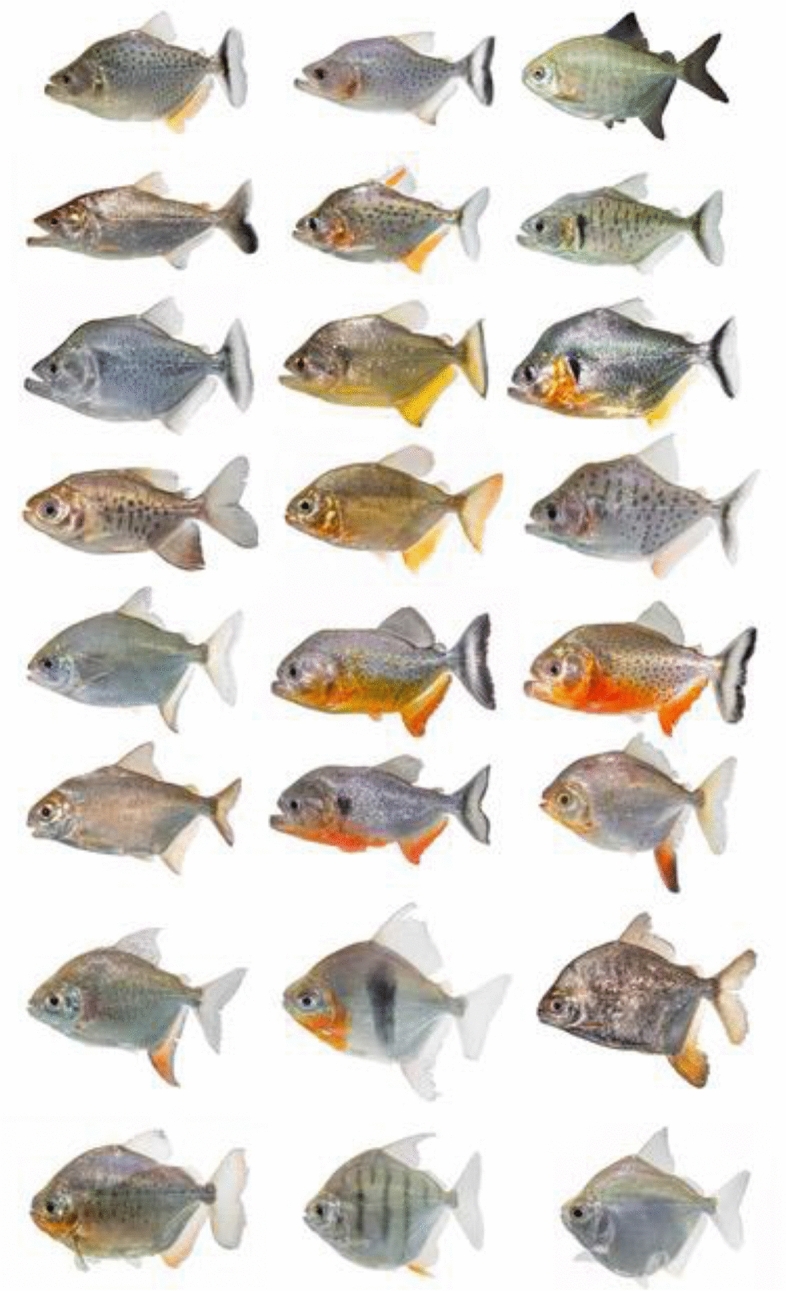
Example of serrasalmid diversity from seven regions: Amazon Lowlands, Orinoco, Pantanal, Tapajos, Tocantins, Araguaia, and Xingu. Species shown include both piranhas and pacus. Species names omitted to illustrate the challenge with identification due to the similarity among species. Photographs taken under standardized conditions as white screen photographs which emphasize the color and pattern of each taxon. Under non-ideal conditions of observation species are more difficult to differentiate (see Figure S7).

Analysis of the naturally occurring variability in the spectral signatures for any particular species suggests that natural historians and taxonomists can use hyperspectral data to discriminate among distinct species or perhaps even different populations of organisms in the field, however the use of hyperspectral data in this way has not been rigorously tested. We remedy this by studying how effective hyperspectral data are in distinguishing taxa along three categorical lines of interest, in particular: (1) phylogenetic and taxonomic relatedness, (2) ecological similarity, and (3) ontogenetic stage. We did this by analyzing the spectral signatures of South American characiform fishes (piranhas and pacus: family Serrasalmidae), as these animals are difficult to distinguish even under controlled conditions (Fig. 1) and because the effort of sampling for aquatic organisms is typically more difficult than terrestrial or even volant species, which are more easily observable. We hypothesize that each species will have its own unique deterministic spectrum, making this metric suitable for rapid species identification in the field as well as elsewhere (markets, aquariums, aquaculture, commercial fishing, etc.). Analysis of the spectral signatures appears robust to inter-individual variation, assuming that organisms are not damaged in anatomical regions of interest.

Why use pacus and piranhas (Serrasalmidae) as a case study for surveying spectral signature diversity? The serrasalmid fishes hold important ecological, economic, and cultural significance in South American river basins and yet are plagued by centuries of taxonomic uncertainty. This system is particularly appropriate system for demonstrating how new methods of rapid field identification can improve our understanding of biodiversity at local and regional scales. Attempts to reconcile taxonomic uncertainty and bring about an evolutionary understanding of the family through molecular methods have corroborated the dubious taxonomic past of the family and continue to find issues with morpho-taxonomic genera designations. Currently, there are about 40 species of piranhas in five genera and 60 species of pacus in eleven genera, found in nearly all habitats in South America^27^. Around half of serrasalmid genera are non-monophyletic (*Tometes, Mylesinus, Myleus, Myloplus, Serrasalmus* and *Pristobrycon*) while the remainder are less speciose or monotypic (*Piaractus, Mylossoma, Colossoma, Acnodon, Ossubtus, Pygocentrus, Catoprion,* and *Pygopristis*)^28,29^. Within these genera, recent barcoding efforts suggest that some 30% of lineages harbor cryptic diversity or undescribed species^30^. Moreover, serrasalmids and many other tropical fishes are under significant pressure from overfishing, pollution, and especially from habitat loss or alteration. Dams, local and global climate change-induced drought, and changes in water conditions (turbidity, pollution) may affect the sensitive balance needed for plant and animal communities to thrive, particularly primary mesopredators and grazers like serrasalmid fishes^31^.

## Results

### Spectral measurements

**Figure 2:**
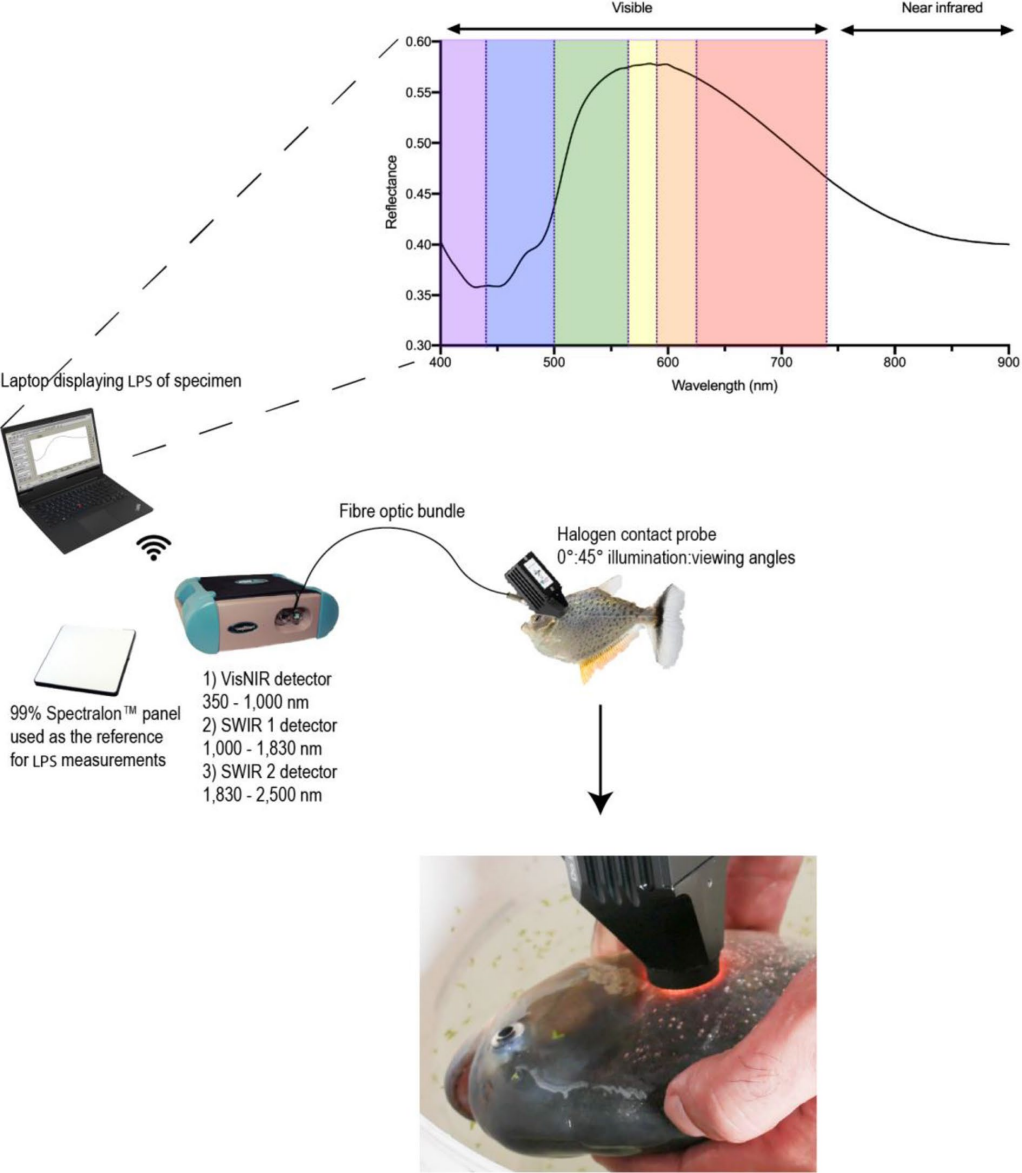
ASD Fieldspec 3 spectroradiometer with fish during measurement. Inset shows a typical spectral signature included in the analyses from 400 to 900 nm: the wavelength range collected by both models of spectroradiometers. The visible wavelengths are shown based on the ‘color’ they represent along with the near infrared region at longer wavelengths. Photograph shows a *Pygocentrus nattereri* being measured at the Laboratório de Ictiologia do IMASUL in Campo Grande, Brazil.

**Figure 3:**
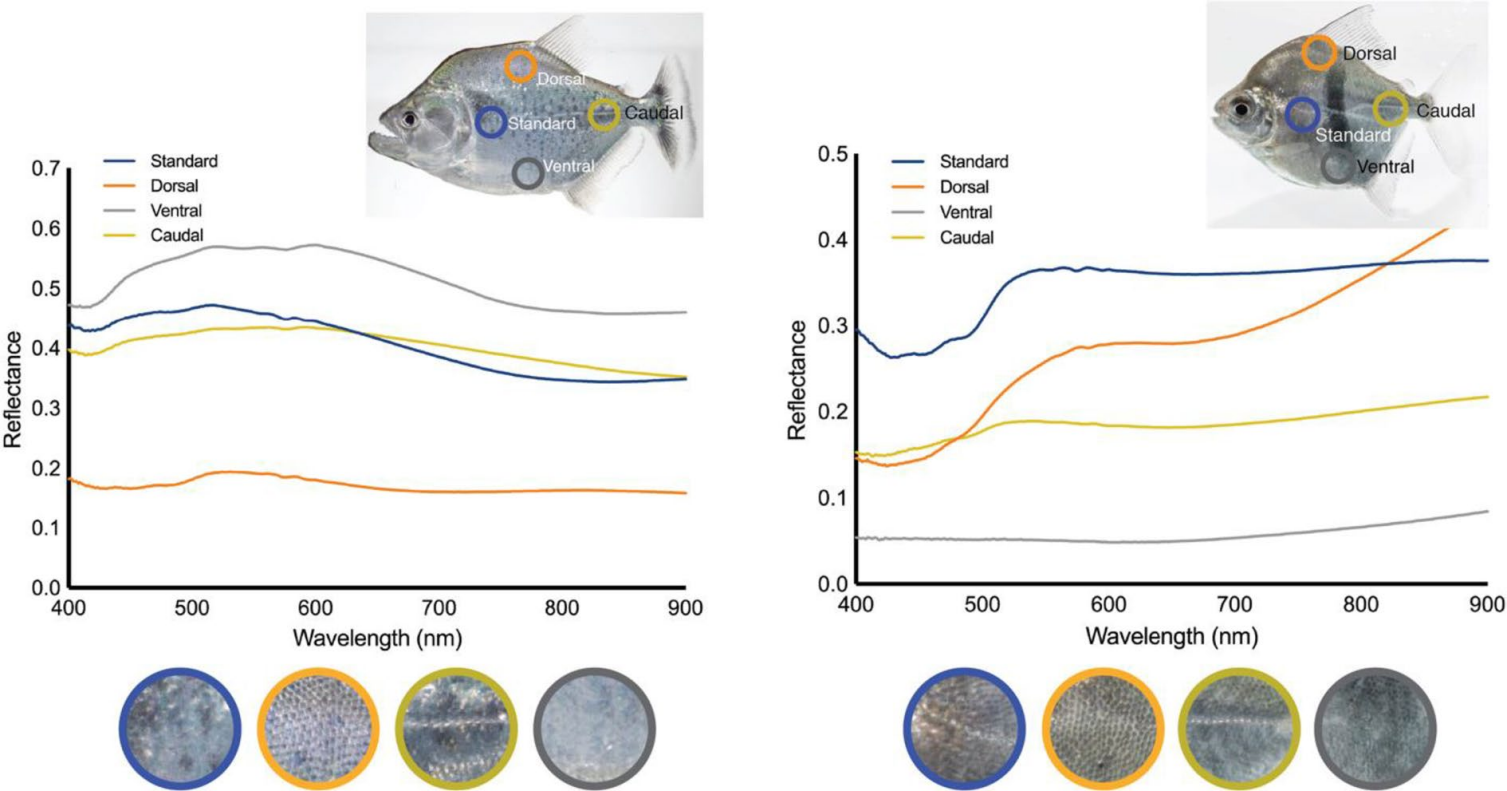
Comparative spectral signatures from four different body regions of a pacu (*Myloplus schomburgkii*) (right) and a piranha (*Serrasalmus geryi*) (left). The four lines correspond to the spectral signatures from the four different locations on the body where the measurements were made. The circular cut-outs illustrate a close-up view of the portion of the fish contributing to each spectral signature. Visually there is a high degree of similarity in the close-up view of the 0.79 cm^2^ (1 cm diameter) spot sizes of the same location between the pacu and piranha (i.e., RGB color, texture, pattern), however, the spectral signatures between these two species are different at each location measured on the body.

To standardize our sampling procedure and reduce measurement error, we assessed how spectral signatures varied across regions of the body in serrasalmid fishes. The spectral signatures of fishes varied depending on where the probe was placed (Figs. 2, 3), as fish body shape also varies greatly across clades^32^. For comparative purposes, it is therefore important that the probe is always placed in a standard location (Fig. 2). In Fig. 3, we show an example pacu (*M. schomburgkii*) and piranha (*S. geryi*), which demonstrate that the spectra differ in both spectral shape and amplitude (i.e., brightness) between species, but also within the same individual (depending on probe placement). Although close-up photographs show similarities among species at each given sampling location, there can be marked differences in spectra. For example, the caudal spectrum has a different shape between the two species, but examination of the photograph illustrates that visually they are similar in color, texture, and pattern. Similarly, the dorsal spectra are different in shape despite similarity in the scales, with the reflectance increasing to 40% on the pacu at wavelengths longer than 700 nm, whereas for the piranha the reflectance is constant at ~ 18% in that same wavelength range.

### Spectral dissimilarity

**Figure 4:**
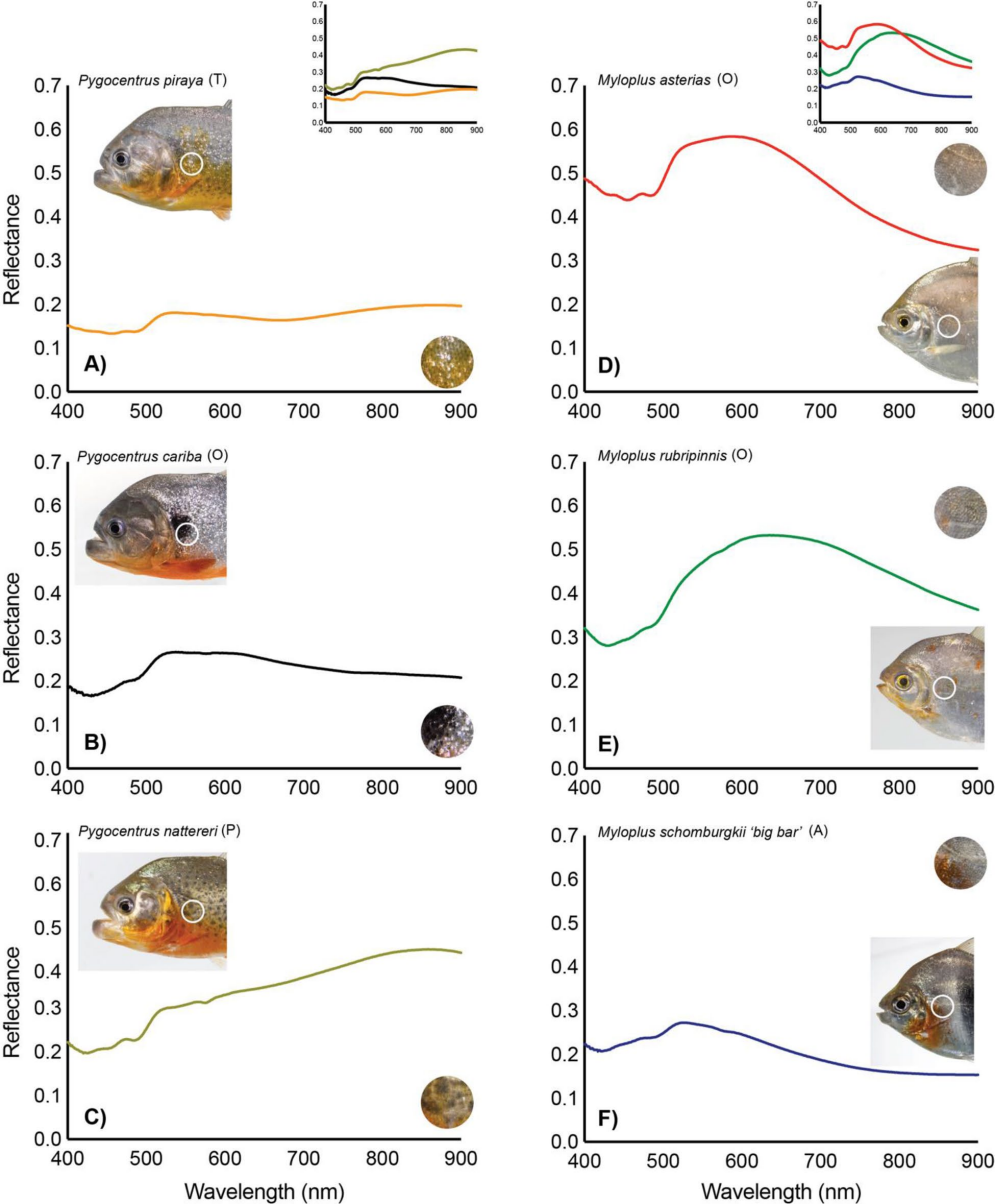
Comparative spectral signatures from three piranha (**a**–**c**) and three pacu (**d**–**f**) species. The circular cut-outs illustrate a close-up view of the portion of the fish contributing to each measurement. Differences among species' spectral signatures can either be changes to the shape of the curve (wavelength) or shifts in the amplitude (brightness).

We demonstrate that when spectra of individual fishes are matched to spectral libraries (i.e., collection of spectra of different species), these individuals can be distinguished from related species, as well as from conspecifics at different ontogenetic stages and from different geographic regions. Our measurements indicate that spectra vary in shape across the 79 samples (Fig. 4). The mean α from the all-pairwise comparison ranges from 0.13 ± 0.09 (*Serrasalmus elongatus*, adult, Amazon lowlands) to 0.53 ± 0.12 (*Piaractus mesopotamicus*, Pantanal). The smallest spectral angle (α = 0.011) was found between a piranha, *Serrasalmus odyssei* (Amazon lowlands), and a pacu, *Colossoma macropomum* (juvenile, Amazon lowlands). The largest spectral angle (α = 0.688) was also found between a piranha *Serrasalmus geryi* (juvenile, Araguaia) and a pacu, *P. mesopotamicus* (Pantanal). Overall, samples with the most distinctive spectra (i.e., largest α) were found to be *P. mesopotamicus* (Pantanal), *Pygocentrus nattereri* (Araguaia) and *P. nattereri* (Amazon lowlands) with 93.7, 92.1 and 86.7% of α > 0.3. Species with the least distinctive spectra (i.e., lowest α) were found to be *Serrasalmus hollandi* (Amazon lowlands) and *Myloplus schomburgkii ‘big bar’* (Xingu) with 46% of α < 0.1.

**Figure 5:**
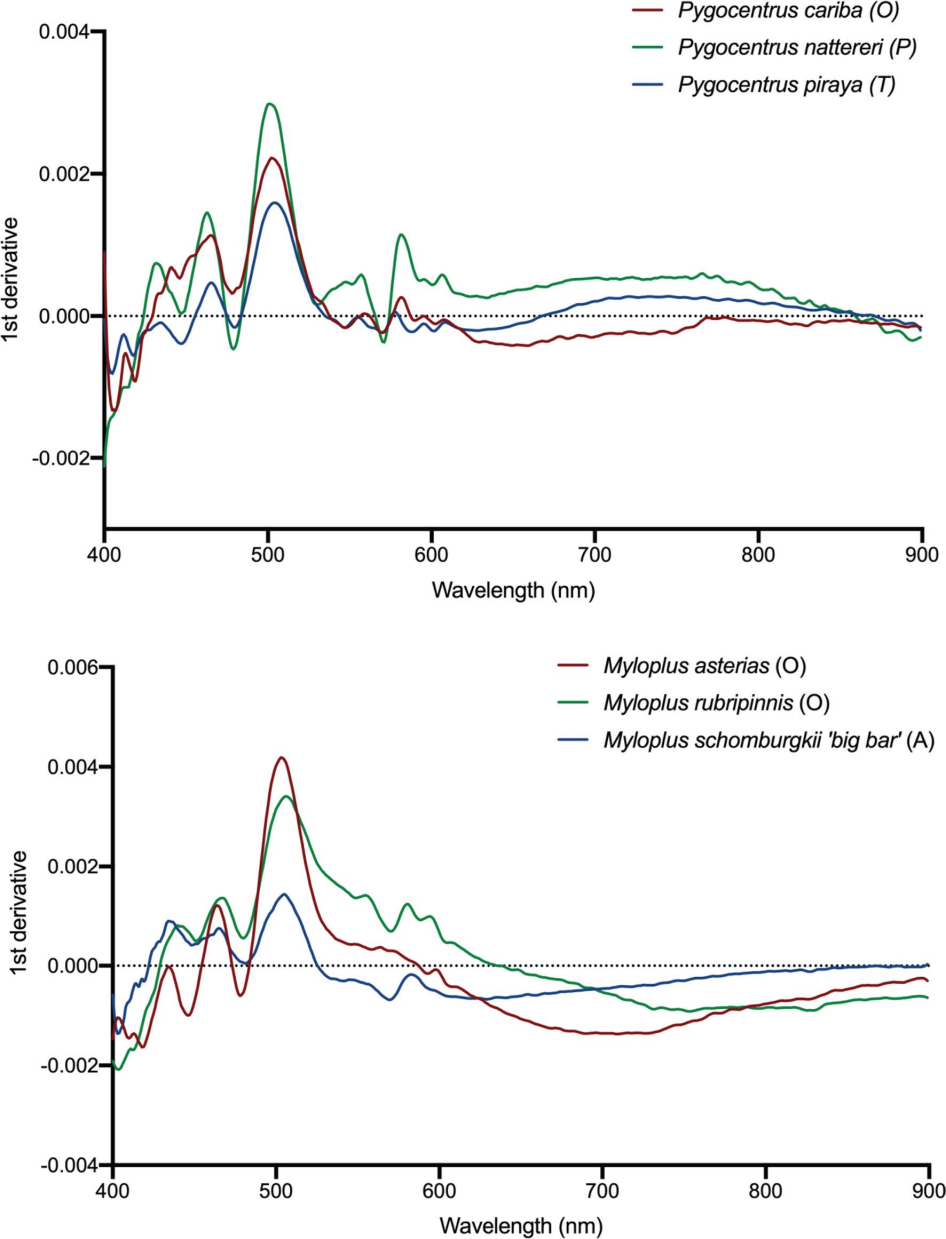
First derivative of the spectral signatures from three piranha (**a**–**c**) and three pacu (**d**–**f**) species shown in Fig. 4.

Figure 4 illustrates the spectra for three piranhas from the genus *Pygocentrus* (i.e., *P. cariba, P. piraya and P. nattereri*) and three pacus from the genus *Myloplus* (i.e., *M. asterias*, *M. rubripinnis*, and *M. schomburgkii* ‘*big bar*’). Despite close phylogenetic relationship between these taxonomic species of the same genera, the spectra vary in shape between each species. This difference is accentuated by the first derivative (Fig. 5) which illustrates the wavelengths at which local minima and maxima are found (i.e., absorption features and peaks). Between the three piranhas, the greatest difference in the first derivate of the spectra can be seen in the 400–480 nm and 530–570 nm ranges. Between the three pacus, the first derivative of spectral signatures shows the greatest difference in the 400–460 nm and 530–610 nm ranges.

The hierarchical clustering of α (Figure S1) indicates that there are three clusters, each with piranha and pacu samples. A broad range of taxonomic species are grouped together in each cluster. However, samples representing the same taxonomic pacu species from different locations are in the same cluster except for *Tometes ancylorhynchus* (Xingu and Araguaia), which has notable color differences among basins and the juvenile specimen of *Myloplus asterias* (Araguaia) in cluster 1 is separated from the adult specimens from the Araguaia, Tapajos, Orinoco, and Xingu rivers all in cluster 2. However, the fidelity of clustering is more variable for piranhas. Adult and juvenile samples cluster together (*S. elongatus, S. geryi, S. humeralis*) but samples from different locations vary across clusters (e.g., *S. rhombeus* (P) is in cluster 3, *S. rhombeus* (A) is in cluster 1). For other piranhas (e.g., *S. altuvei*) samples from different locations cluster together.

**Figure 6:**
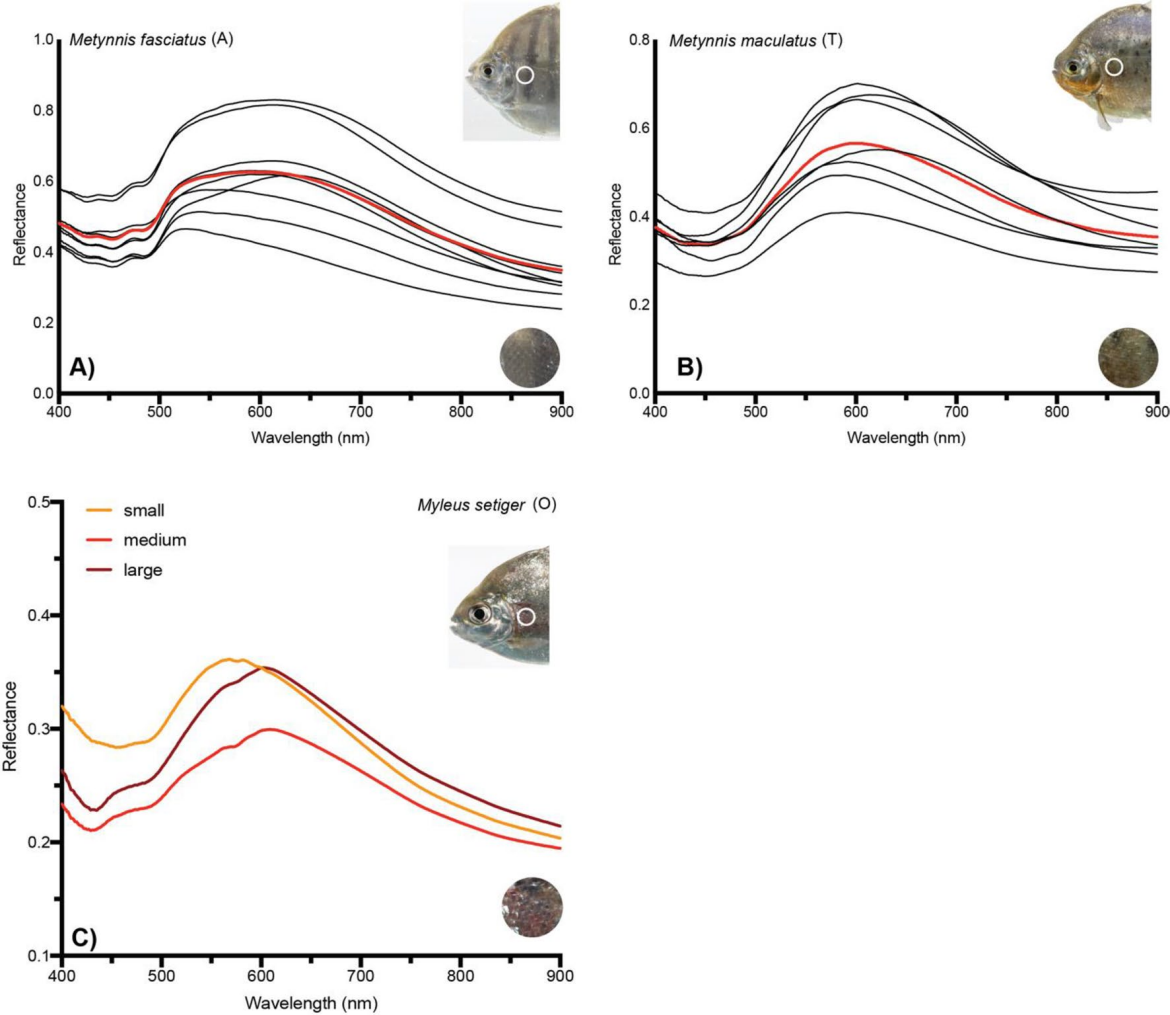
Intraspecific spectral signatures comparisons within select serrasalmid taxa. Variation within (**a**) *Metynnis fasciatus* and (**b**) *Metynnis maculatus*. Each line represents the spectral signature of an individual. Mean spectral signature is shown in red. (**c**) Ontogenetic change in spectral signature variation in *Myleus setiger*. Inset photograph shows a ‘medium’ individual. The shift in peak spectral signature towards red wavelengths can be seen from medium and large individuals. Circular close-up of the measurement area shows a red coloration of the scales. This is absent in small individuals.

**Figure 7:**
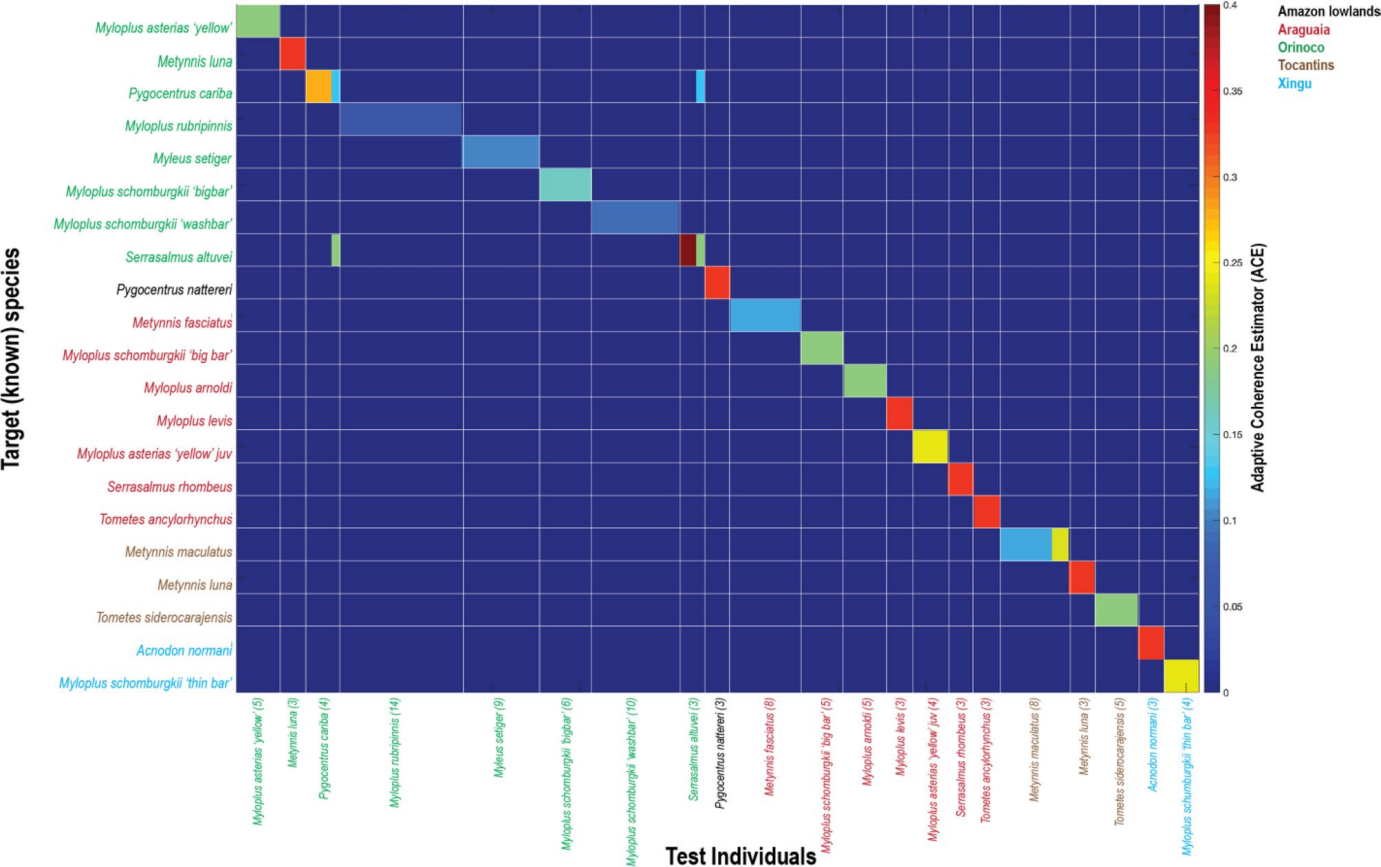
Adaptive Coherence Estimator (ACE) spectral signature ‘matching’. Taxa on the x axis indicate the target, or ‘known’ species. Taxa on the y axis are the test individuals that are matched to the most similar ‘known’ species through ACE. The numbers in brackets indicate the number of individuals of a given species included in the test set. Each specimen is matched individually. Higher values of ACE indicate a stronger match. Although the strength of the ACE match can vary within species (e.g., *Metynnis maculatus*), a species' ACE value is shown to be more like its own species than even close relatives. The taxa with the lowest ACE matches are typically those with the broadest geographical ranges (e.g., *Myleus setiger, Myloplus rubripinnis, Myloplus schomburgkii ‘*wash bar’ Orinoco from the *M. schomburgkii* species complex) and therefore the greatest potential for cryptic lineages through low population connectivity.

The spectra of multiple individuals from the same sample (e.g., *Metynnis maculatus* (T)) are consistent in shape (Fig. 6). Amplitude variability can be seen between individual spectra but the overall shape within a species is more similar than among species (Fig. 7). This suggests that although spectral signatures are sensitive enough to detect interspecific plasticity, the plasticity itself is not stochastic enough to preclude species identification. The fishes with the weakest ACE match (*M. setiger, M. rubripinnis, M. schomburgkii*) are pacus (subfamily Myleinae) that are some of the most phenotypically variable across broad geographic regions. In contrast, species from the subfamily Serrasalminae (*P. nattereri and M. luna*) which are also phenotypically variable across broad geographic regions are amongst the strongest ACE matches. For samples with multiple individuals, ACE identified the correct known species (Fig. 7). The strength of the match varied from *Serrasalmus altuvei* (O) with ACE = 0.4 to *Myloplus rubripinnis* (P) with ACE = 0.06 with all other non matches across samples at 0–0.001. For one *S. altuvei* (P) individual, ACE returned a second lower potential match to *Pygocentrus cariba* (P) ACE = 0.13 (in contrast to the 0.3 for the correct match). Similarly, for one *P. cariba* (P) individual, ACE returned a second lower potential match to *S. altuvei* (P) ACE = 0.13 (in contrast to the 0.28 for the correct match).

The four phenotypic varieties of the *M. schomburgkii* species complex (Figure S2) show variability in spectra, indicating the importance of regional differences. The spectra suggest at least two cryptic species among the specimens measured. The most similar spectra (α = 0.046) were found between *M. schomburgkii* ‘thin bar’ (X) and *M. schomburgkii* ‘wash bar’ (O). The most different spectra (α = 0.182) were between *M. schomburgkii* ‘big bar’ (A) and *M. schomburgkii* ‘big bar’ (O). Consistently, *M. schomburgkii* (O) had largest spectral angle in comparison to the other three varieties indicating it is the most unique spectrum of the four. All individuals of the four varieties were correctly matched to the correct label through ACE (Fig. 7).

General patterns of spectral separability between piranha and pacu lineages indicated an optimal 38 bands (Figure S3) concentrated in the 400–450 nm range and 525–560 nm (Figure S3) reaching a nearest neighbor criterion value of 0.85 (max = 1). In contrast, 58 bands were required for optimal differentiation between subfamily classes concentrated in the 450–525 nm range. These ranges are like the regions highlighted by the first derivative analysis (Fig. 5) differentiating between the three piranha and pacus species. All four classification methods indicated a similar accuracy for separating between piranha and pacu lineages with a best result of 81.3% (linear discriminant). Overall, there was lower separability between subfamily classes with a maximum nearest neighbor criterion value of 0.67 (Figure S3). The resultant classification accuracy of subfamily classes also decreased with a best result of 68.8% (neural network). The optimal wavelengths separating subfamily classes are clustered in the same wavelengths of sensitivity (λ_max_) in fish vision^33^ from all five levels of chromacy (monochromacy–pentachromacy) whereas λ_max_ of cones for human^34^, otter^35^ and bird^36^ color vision are more similar to the optimal wavelength regions for differentiating between piranhas and pacus (Figure S4).

### Gross scale morphology

We might suspect that differences in scale morphology might be driving overt distinctions in spectral signature; if so, then we would expect scale morphology to vary or co-vary as substantially among species and clades as spectral signature appears to do. So, we measured scale topology and macrostructure, finding that five of our six selected species have smooth-edged cycloid scales (the exception being *Myloplus schomburgkii*) with typical morphological features such as a curved posterior edge, a highly overlapping pattern, and concentric growth lines (circuli) that form ridges on the surface of each scale (Figure S5). The scales of these fishes are generally small (< 1 mm exposed length) and therefore create surfaces with low roughness (4–14 µm root-mean-square roughness). This low roughness accentuates the directional specular reflective properties of the fishes. Scales from each species have distinct shapes, but all within normal variation between species. *Myloplus schomburgkii* exhibits the largest difference, as it has scales with a weakly serrate posterior edge, and therefore we would classify these scales as weakly crenate. *Myloplus schomburgkii* also has a higher roughness than the other species (21–25 µm root-mean-square roughness), which is true even accounting for the larger size and (therefore) scales of the imaged specimens of this species. In short, although there are slight differences among species, the scales of these six taxa have typical cycloid and crenate scales.

### Phylogenetic signal

Finally, we might expect that spectral signature might vary predictably with phylogeny (evolutionary relationships among related species), i.e., that spectral signatures are more similar among sister species, relative to another more distant relative. We trimmed a published molecular phylogeny (from Thompson et al.^29^) to the 34 terminal taxa that we were able to measure spectral signature from (Figure S6). Results for phylogenetic signal show no correlation between phylogeny and composite spectral signals as represented by our first two PC axes (PC1: *K* = 0.163, p = 0.375, λ < 0.0001, p = 1.0; PC2: *K* = 0.158, p = 0.467, λ < 0.0001, p = 1.0). Estimates of lambda (*λ*) range between zero (no correlation between species) and 1.0 (some correlation between species), relative to a Brownian Motion (random motion) expectation for trait evolution. Conversely, *K* roughly approximates how trait variance is distributed along the phylogeny: if *K* > 1 then variance tends to be among clades, while *K* < 1 suggests variance is within clades. There appears to be no phylogenetic signal to spectral data for this group, suggesting perhaps that these values evolve in a random fashion, or according to environmental factors we cannot yet identify.

## Discussion

Consistent differences among species’ spectral signatures confirms that we can use hyperspectral data to discriminate among different fish species, as demonstrated with pacus and piranhas as a proof-of-concept. Species generally differed from one another in their spectral signatures in accordance with changes to either spectral shape and/or amplitude. The application of this method to different aspects of biodiversity and natural history research is considerable, as spectra can be used to: (1) rapidly assess lineage diversity along field transects, (2) act as a ‘first-pass’ screening method for new species delimitation, and as (3) a potential means for assessing species *en masse*, e.g., using remote sensing coupled with machine learning as a tool for better management of ornamental fish exportation. Moreover, since the basis of a lineage’s spectral signature relates to phenotypic aspects of scale morphology and pigmentation^37–41^, this method serves as rapid, morphology-based means of assessing biodiversity like how DNA barcoding complements other molecular methods in phylogenetics, population demography, and community ecology. With biodiversity losses at an all-time high, we believe that spectral characterization of distinct lineages will aid traditional morphological taxonomy and molecular methods of surveying biodiversity.

We also highlight differences in the spectra within lineages that may be of interest to taxonomists: *Myloplus schomburgkii*, for example, appears to harbor several distinct lineages that equate with different rivers across their large range. This correlates with observations on the ecological and phenotypic differences among populations of *M. schomburgkii*, as well as documented differences in the population genetics of this species complex^42^. In accordance with the findings of Machado et al.^30^, we document distinctive *M. schomburgkii* lineages from the Xingu River and Araguaia Rivers, which are in turn distinctive from an Orinoco/Guiana Shield lineage. This method’s ability to discriminate among juveniles and adults, as well as tease apart morphological patterns among species complexes, will aid taxonomic descriptions and re-appraisals in addressing ontogenetic changes within certain lineages, an issue that continues to plague morpho-taxonomy.

What defines the spectral signatures of a given species? It appears as if phylogenetic relatedness does not overtly contribute to spectral signature variation as much as physiological or ecological differences in habitat may; there are differing selective regimes acting on the visual ecology of fishes living in different microhabitats^43^ or in rivers with different water types: blackwater, whitewater, and clearwater (Figure S7). Convergence along biologically relevant regions of the visual light curve in different species suggests a burgeoning area of research for those interested in camouflage^24,25^, crypsis^44^, intra- and/or inter-specific signaling^45,46^. As such, we demonstrate that although individuals are near identical at distinct size classes for example, we see changes to the spectra of species as they navigate ontogeny (Fig. 6). It is difficult to distinguish among the sexes in many Neotropical fishes, but it is quite possible, especially given seasonal dimorphism in fishes like pacus^47^, that spectral signatures can be used to investigate visual evolution and signaling as they pertain to sexual selection in fishes.

We live in a time of biodiversity crisis, particularly when institutional funding for conservation and environmental sustainability are limited. Lean times require prioritization of resources and before anything else, an accurate, comprehensive catalog of our living resources^48^. One of the concerns with DNA barcoding technologies is that they can precipitate a scenario where biodiversity is quantified but never described (but see Packer et al.^7^). Measurement of spectral signatures offers a rapid, intuitive method for quantifying phenotypic diversity in the field or lab and furthers inclusivity, digitization, and coordination in natural history collections, and can help morphological taxonomists delineate cryptic lineages^49–52^. In particular, the spectra do not serve as an alternative to or discourage the collection of physical specimens, but instead should help clarify which specimens might be the most unique^48^. Considering that not all taxonomic efforts take place immediately after field expeditions, but in many cases, years or decades later when specimens are re-examined^53^; our next effort is to determine how the preservation process alters the spectral signature of specimens. Therefore, we propose that reconciling historical museum collections with cutting-edge remote sensing efforts will streamline and expand the international scientific community’s ability to assess biodiversity and prioritize taxonomic efforts along shorter timescales.

One of the benefits of other rapid diversity assessment tools like DNA barcoding and metabarcoding is the efficiency of such methods in performing broad, community-level sampling. A common barrier to researchers interested in applying new methods like ours, is cost. Portable spectroradiometers suitable for field conditions range anywhere from $15,000–20,000; however, these devices incur a one-time cost without consumables. Stein et al*.*^54^ estimated the cost of DNA barcoding methods relative to traditional taxonomic evaluations of field diversity and found that both Sanger and next-gen sequencing platforms were one and a half to three times as expensive as morphology-based taxonomy. As opposed to both traditional morphological approaches and molecular methods, the spectral signature method allows for biodiversity assessment almost instantaneously in the field. Field work requires additional funding burden on other methods of taxonomic cataloging^54^, whereas these costs are integrated into the operation of our spectral signature method.

We informally compared the start-up costs for DNA extraction and amplification equipment needed for DNA barcoding (while considering that actual sequencing would be outsourced to dedicated sequencing facilities). We suggest that the start-up costs for barcoding or our spectral signature method would be similar (mean cost for barcoding estimated below as $17 k, while high quality, well characterized portable spectroradiometers start at $15 k), albeit for molecular techniques, costs are spread out across individual machines, reagents, etc., whereas purchasing a spectroradiometer is a one-time expenditure. Stein et al.^54^ estimated the operating costs for DNA barcoding (i.e., once a working lab is established), to be around $5 USD per sample or about $1500 for a 200-sample plate. These consumable costs are largely avoided with spectroradiometry. Another major difference in cost/effort between Sanger sequencing and spectroradiometry is the possibility for PCR reactions to fail to amplify or amplify the wrong loci or non-overlapping sections of the same loci. While this hurdle necessitates some tweaking of primers and thermocycler routines, when a fish moves during the acquisition of its spectrum, the 10 s procedure is simply repeated, whereas redoing PCR reactions requires several hours before results can be visualized again and checked for quality. Like molecular barcoding results, early processing and analysis for spectral data requires detailed training, but because the output from most spectroradiometers are standard ASCII files which can be read with most analytical software (e.g., Excel, R, MATLAB, etc.), these methods are not any more enigmatic (perhaps even less so), than the sort of training that goes into interpreting chromatograms, aligning sequences, and running phylogenetic or phenetic analyses on molecular data. Hardware for acquisition of spectra has an initial start-up cost, but that is comparable to established morphological and molecular taxonomic methods in the long term.

Moving forward, questions remain about spectra and their use for biodiversity research in ichthyology. For example, what specific aspects of scale morphology and pigmentation, both microstructural and biochemical, are shaping differences among species’ spectral signatures? Modern teleost scales are composite structures of laminate sheets of cellular and acellular bone (predominantly the latter). The hydroxyapatite structure of these scales can reflect and refract light given differences in thickness, density, and material composition. The interaction of light moving through these bony layers with the underlying pigment (not to mention overlying mucus coating of fishes), is rife with research possibilities^55,56^. Surely the visual environment of different riverine habitats promotes altering selective regimes on coloration through changes to pigment and scale phenotypes.

## Methods

### Spectral measurements

Measurements from live animals took place at a commercial facility (BelowWater, Montreal, Quebec), on site in Brazil (Salobra river), at the Laboratório do Ictiologia de Altamira, Federal University of Para in Altamira, Brazil and the Laboratório de Ictiologia do IMASUL in Campo Grande, Brazil. Specimens measured in Brazil were collected under permit SISBIO 31089-2 and in Canada animals were handled by staff from the commercial facility (BelowWater, Montreal, Quebec) and according to the guidelines and regulations of McGill University (Montreal, Quebec, Canada), no separate animal care certificate was required. We measured the spectral signatures of live fishes from 47 species (27 pacus, 20 piranhas) representing all 16 serrasalmid genera (Fig. 1, Table S1), originating from river systems in Brazil, Colombia, Peru, and Suriname. Twelve of the species were obtained from multiple river systems and each was treated as a unique sample in our analysis. Six of the species were available in two sizes (adult and subadult) which were also kept separate in the analysis. In total, 79 unique samples were retained. Thirty-six of the unique samples were represented by multiple individuals. For the other species it was not possible to obtain multiple individuals. A total of 176 fish were measured, ranging from 2.0 to > 30 cm in standard length (SL). We purposely chose to use specimens from the pet trade, for which we also had accurate locality collection data, as well as specimens measured on site in Brazil in Pantanal and in the Xingu to demonstrate the utility of spectral signatures in discriminating among species. Specimens were identified according to traditional meristics and morphometrics^57–59^, and when possible, through next-gen sequencing technologies^60^.

The samples in Canada were measured with an Analytical Spectral Devices (ASD) Fieldspec 3 spectroradiometer (Malvern Panalytical, Boulder CO) (Fig. 2). This instrument measures reflected radiation in the 350–2500 nm range. It has a spectral resolution of 3 nm and a sampling interval of 1.4 nm in the visible and near infrared regions (Vis–NIR) and a spectral resolution of 10 nm with a sampling interval of 2 nm in the shortwave infrared (SWIR). The instrument is composed of three separate detectors, a 512-element silicon photo-diode array for the Vis–NIR and two graded index, TE-cooled, extended range, InGaAs, photo-diode detectors for the SWIR 1 (1000–1830 nm) and SWIR 2 (1830–2500 nm). The samples in Brazil were measured with an ASD Handheld spectroradiometer (Malvern Panalytical, Boulder CO). It measures reflected radiation in the 325–1075 nm wavelength range. The instrument has a spectral resolution of 3 nm and a sampling interval of 1.6 nm. The single detector is a 512-element silicon photo-diode array. The field of view of the fiber optic cables of both instruments is 25°.

A contact probe with a low intensity halogen light source was used for all samples (Fig. 2). The probe ensures a constant illumination and viewing geometry with a 1 cm diameter spot size (sampling diameter). Fishes were removed from the holding aquarium with a nylon mesh aquarium net, and then placed gently on a worktable, while still in the net. All measurements were taken on the left side of the fish with the probe placed on the fish’s flank directly posterior to the operculum (Fig. 2). The pectoral fin was moved out of the way so that the probe was only illuminating scales. The probe was placed flat against the body of the fish and its lens was wiped clean between fish, to reduce water droplets or fish slime. Each recorded sequence took 8–12 s to complete, resulting in less than 30 s that each specimen was out of water, after which each specimen was returned to its aquarium. Measurement of a standard location posterior to the operculum was chosen, because serrasalmids (and many other fishes) are generally flat in this region and setting the front edge of the probe against the operculum makes for a tactile cue to consistently measure the same location on multiple individuals. Due to the illumination and viewing geometry of the probe, facing the fibers with the viewing angle towards the caudal fin mitigated detector saturation from the directional specular reflectance properties of the scales^61,62^.

All measurements were made in relation to 99% reflective Spectralon panels which had been characterized to a NIST traceable standard. The reflectance ratio acquired with both instruments were processed to estimated absolute reflectance using the methodology described in detail by Elmer et al.^63^. The estimated absolute reflectance is independent of illumination intensity or conditions and is recommended for comparisons of spectra even if collected by different sensors, or with different reference panels^63^. The values of reflectance in the 400–900 nm range common to both instruments were retained for analysis.

For the samples where multiple individuals were measured, a representative spectrum was chosen as the individual spectrum with the smallest spectral angle (α) to the mean (Eq. 1)^64^,

1 $$\alpha ={\cos}^{-1}\frac{\Sigma XY}{\sqrt{\Sigma {(X)}^{2}\Sigma {(Y)}^{2}}}$$

where X is the mean spectrum across the 400–900 nm range and Y is an individual spectrum across the same range. The spectra angle, α, is expressed in radians in *n* dimensions between the two signals (in this case n = 501). Selecting a representative spectrum preserved spectral features inherent in the spectral variability that may have been lost because of averaging^65^. This measure of similarity between two spectra is insensitive to differences in the magnitude of brightness^64^, which can be affected by the sliminess or moistness of the fish’s skin and scales. Smaller spectral angles (α) indicate spectra that are more similar. The mean spectrum per sample was also retained as a generalized spectral signature for samples with multiple individuals.

### Spectral dissimilarity analyses

Due to high dimensionality of hyperspectral data, its greatest strength is in applications where the spectral information (i.e., reflectance and absorbance) is more informative or reliable than the morphology of the object under study^66^. However, due to the relatively small sample size (i.e., few individuals) for many of the samples, conventional classification algorithms for grouping similar spectra (e.g., classifying species) are not applicable to this dataset. The number (n) of spectra per species/sample is too small to estimate the statistical properties of the target class (i.e., species of serrasalmid) from the full set of spectra acquired. Therefore, in examining the separability of the spectra between samples, we conducted our analyses following the hyperspectral target detection literature for which our sample size is adequate.

First, the spectra of all 79 samples were analyzed according to an all-pairwise comparison of α between samples based on the representative spectrum. To visualize potential clusters of sample pairs with low $\alpha$, indicating spectral similarity, a hierarchical cluster analysis was performed using the centroid as the method for the distance calculation. With the centroid, the distance between two clusters is the squared Euclidean distance between their means, meaning this method is more robust to outliers than other hierarchical methods.

Next, for a subset of 21 samples from four river basins which had a minimum of three individual specimens per sample, a spectral matching analysis was carried out using the Adaptive Coherence Estimator (ACE) (Eq. 2), a standard benchmark hyperspectral detection algorithm^67^:

2 $$ACE = \frac{{({S}^{T}{\Sigma }_{b}^{-1}x)}^{2}}{({S}^{T}{\Sigma }_{b}^{-1}S)({x}^{T}{\Sigma }_{b}^{-1}x)}$$

where S is the target (i.e., the known spectrum of a sample, in this case the mean), x is the spectrum of each individual specimen under comparison and ${\Sigma }_{b}$ is the covariance matrix of the background. *T* denotes a matrix transposition. The background (${\Sigma }_{b}$) was a subset of the entire spectral database of all individual specimens. For each of the 21 samples under consideration a separate ${\Sigma }_{b}$ was calculated omitting any spectra of that sample to prevent corrupting ${\Sigma }_{b}$^67^; for example, when examining the detectability of *Metynnis luna* (O), ${\Sigma }_{b}$ was calculated with all *M. luna* (O) spectra omitted from the database. ACE also considers spectral shape rather than amplitude (i.e., brightness) and can be described as the cosine square of the angle between the test spectrum (x) and the known target subspace (S)^66^. The ACE detector calculates the likelihood that a particular spectrum matches a known sample (S) with higher values indicating a closer match.

Finally, to look for general patterns of spectral separability within serrasalmids, we determined an optimal subset of bands to distinguish between piranha and pacu clades. We also examined the spectral separability of samples that belong to different clades (subfamilies) based on Kolmann et al.^60^. The subfamily classes considered were: (1) Colossomatinae which have a diet predominantly of plant material, seeds and fruits (frugivores); (2) Myleinae which are a mix of granivores (plant material and seeds), and folivorous phytophages (leaves, flowers, stems); (3) the Serrasalminae clade, including the genus *Metynnis*, which are either planktivores or feed on both plankton and plant material; and 4) the piranha branch of Serrasalminae, which feed primarily on fish and fish parts (scales, fins, mucus), including piscivores and several omnivores (plants and fish, e.g. *Pristobrycon striolatus*). The optimal bands were determined through a forward feature selection (FFS) decreasing the total 501 dimensions (bands) to a smaller subset which maximizes separability between the classes of interest^68^. Dimension reduction is important with hyperspectral data because there is redundancy in information across all bands. It is also necessary to reduce the number of training samples for classification (i.e., Hughes phenomenon) to increase classification performance and decrease computational complexity^68^. The result of an FFS is different from transformations such as Principal Component Analysis (PCA) because FFS selects components (i.e., bands) from the full spectra and the original units and meaning of the data (i.e., reflectance) are retained. By grouping the samples into broader categories (e.g., piranha vs pacu) and reducing the number of bands through FFS we increased our samples size with respect to the dimensionality of the data and therefore could apply standard classification algorithms to examine how well the broad categories could be differentiated from the spectra. We trained a linear discriminant classifier, a quadratic classifier, a k-nearest neighbor classifier and a feed-forward neural network classifier. For each of the two scenarios (piranha vs. pacu and subfamily class) the classifiers were trained on a subset of 60% of the spectra with a reduced number of bands based on the FFS and tested on the remaining 40%.

### Morphological and phylogenetic correlates of spectral signatures: profilometry imaging and comparative methods

What aspects of morphology and evolutionary history relate to the spectra measured from serrasalmid fishes? We used gel-based profilometry imaging (GelSight Inc., Waltham, MA, USA) to describe serrasalmid scale shape from a variety of museum specimens. Generally, we assumed that if scale surface texture varies broadly across taxa, this would be reflected in some consistent variability in spectral measures. In brief, gel-based profilometry is a surface imaging technology that combines advantages of optical and contact profilometry to visualize and measure surface topography^69^. It has been previously used in biology to image and measure the surface topography of organisms including other fishes^56^, and it has shown utility in easily resolving features less than 10 µm in height^70,71^ while imaging much larger fields-of-view on the order of 10–300 mm^2^. During gel-based profilometry, the gel is pressed onto the specimen, and the painted side of the gel deforms to match the surface topography of the subject. While the gel is pressed onto the surface of interest, six photographs are taken using six different lighting angles, and these are used to produce a 3D topographical representation of the surface^69^. Additional processing of surface files (cropping, removing body curvature, calculating roughness) is then done in MountainMaps v7.4.8425 (Digital Surf, Besançon, France). We imaged 27 individuals from six species (n = 3–9 per species), all from specimens housed at the Harvard Museum of Comparative Zoology (MCZ) (Table S2).

We were also interested in how much of the spectral variability could be explained by relatedness among serrasalmid lineages. Phylogenetic signal is a measure of the statistical non-independence of related species, i.e., tests of phylogenetic signal determine how much of the observed trait variation can be explained by relatedness among species or groups of species. For the phylogenetic input used for testing phylogenetic signal, we trimmed a recent, comprehensive phylogeny^60^ down to only those species for which spectral data was available and matched these species’ spectra to appropriate tips in the tree. Briefly, this phylogeny is a molecular, time-calibrated chronogram, meaning that many different gene loci were compared, using maximum likelihood methods, to determine the relatedness of each species. The resulting cladogram has branch lengths, which are proportional to the number of changes in nucleotide base pairs, scaled with respect to geological time. We used the reflectance from measured fishes across the Vis–NIR light spectrum (400–900 nm) as our data subset for estimating phylogenetic signal. We used a principal component analysis (PCA) to reduce the dimensionality of our dataset and retained only the first two axes, indicated as the only significant axes by the broken-stick method, for subsequent analyses. Finally, we used two different methods to estimate phylogenetic signal, Blomberg’s *K* and Pagel’s *λ*, and applied these estimates to PC axes through the *phylosig* function (v. 2.0.6.4 *geiger*^70^).

## Supplementary Information

Supplementary Information 1.

Supplementary Information 2.

## Data Availability

Phylogeny materials available here: https://www.biorxiv.org/content/10.1101/2020.03.02.973503v1. HSI data will be archived and made available on Zenodo.
