# Supplementary material for: Hyperspectral data as a biodiversity screening tool can differentiate among diverse Neotropical fishes
Source: Sci Rep. 2021 Aug 9;11:16157. doi: 10.1038/s41598-021-95713-0 (PMC8352966; doi:10.1038/s41598-021-95713-0)
Supplement: Supplementary file 1 — Supplementary Information 1. [file 41598_2021_95713_MOESM1_ESM.pdf]

Hyperspectral data as a biodiversity screening tool can differentiate among diverse Neotropical fishes

Kolmann, M.A.[1*,2] & Kalacska M.[3], Lucanus O.[4], Sousa L. [5], Wainwright D. [6], Arroyo-Mora J.P.[7], Andrade M.C.[8]

Affiliations:
[1]University of Michigan, 1105 N University Ave, Ann Arbor, MI 48109
[2]Royal Ontario Museum, 100 Queens Park, Toronto, ON M5S 2C6
[3]Applied Remote Sensing Lab, McGill University, Montreal, QC H3A 0B9, Canada
[4]Below Water Inc., Vaudreuil-Dorion, QC J7V 0K4, Canada
[5]Laboratório de Ictiologia de Altamira, Universidade Federal do Pará, Altamira, PA, Brazil
[6]Yale University (Peabody Museum)
[7]National Research Council Canada, Ottawa ON
[8] Núcleo de Ecologia Aquática e Pesca da Amazônia, Universidade Federal do Pará, Belem, PA, Brazil

*corresponding author's address:
University of Michigan, Museum of Paleontology, 1105 N University Ave, Ann Arbor, MI 48109
Email: mkolmann@gmail.com

Running headline: *spectral signatures for cataloging biodiversity*

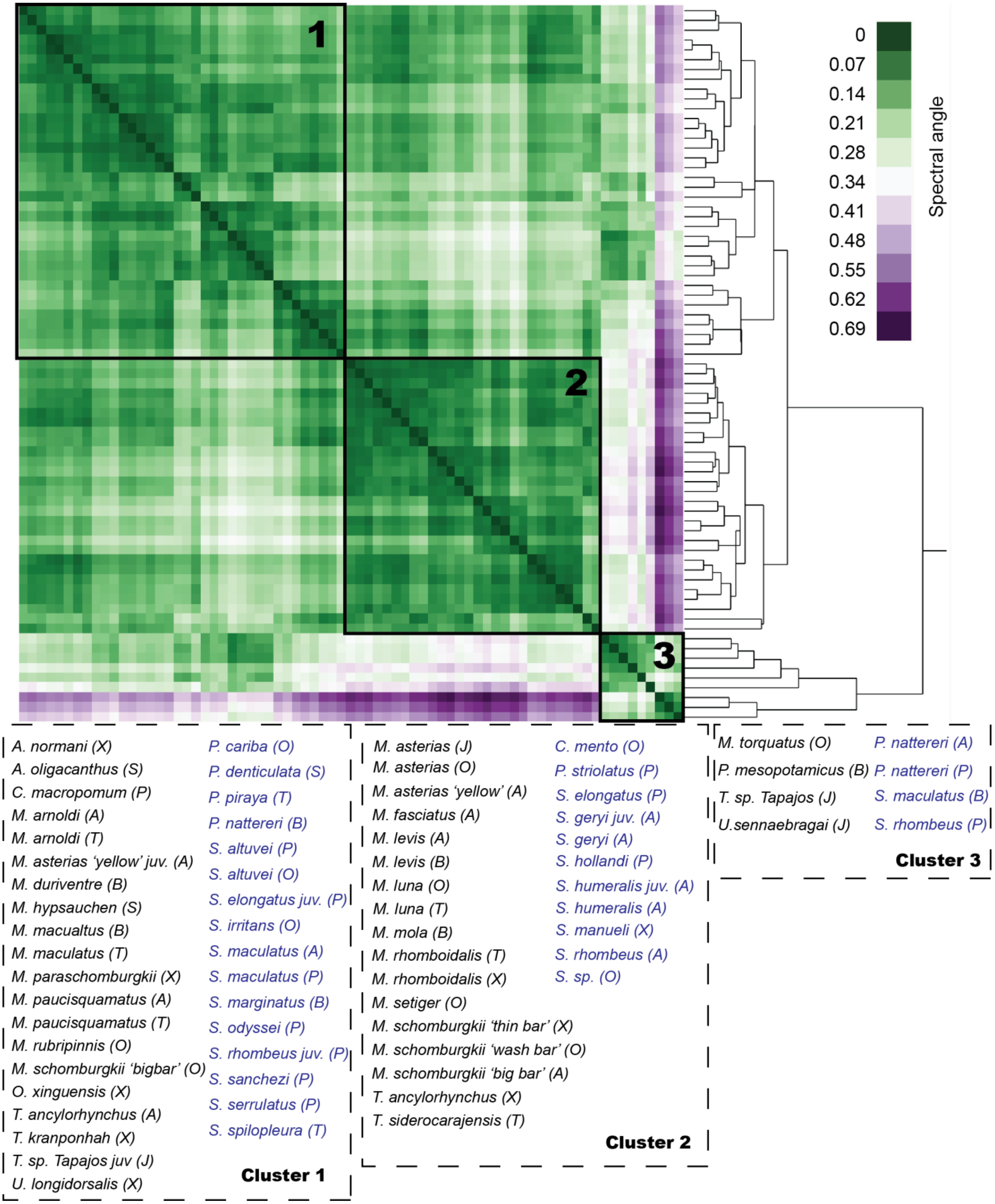

Supplemental Figure S1. Hierarchical cluster results based on the spectral angle between all pairs of samples. Three broad clusters can be seen, containing both pacus (black) and piranhas (blue). The letters indicate the location from where the fish originates: A = Araguaia, B = Pantanal, P = Amazon Lowlands, S = Suriname, J = Tapajos, T = Tocantins, X = Xingu. Smaller values for spectral angle indicate greater similarity among spectral signature values.

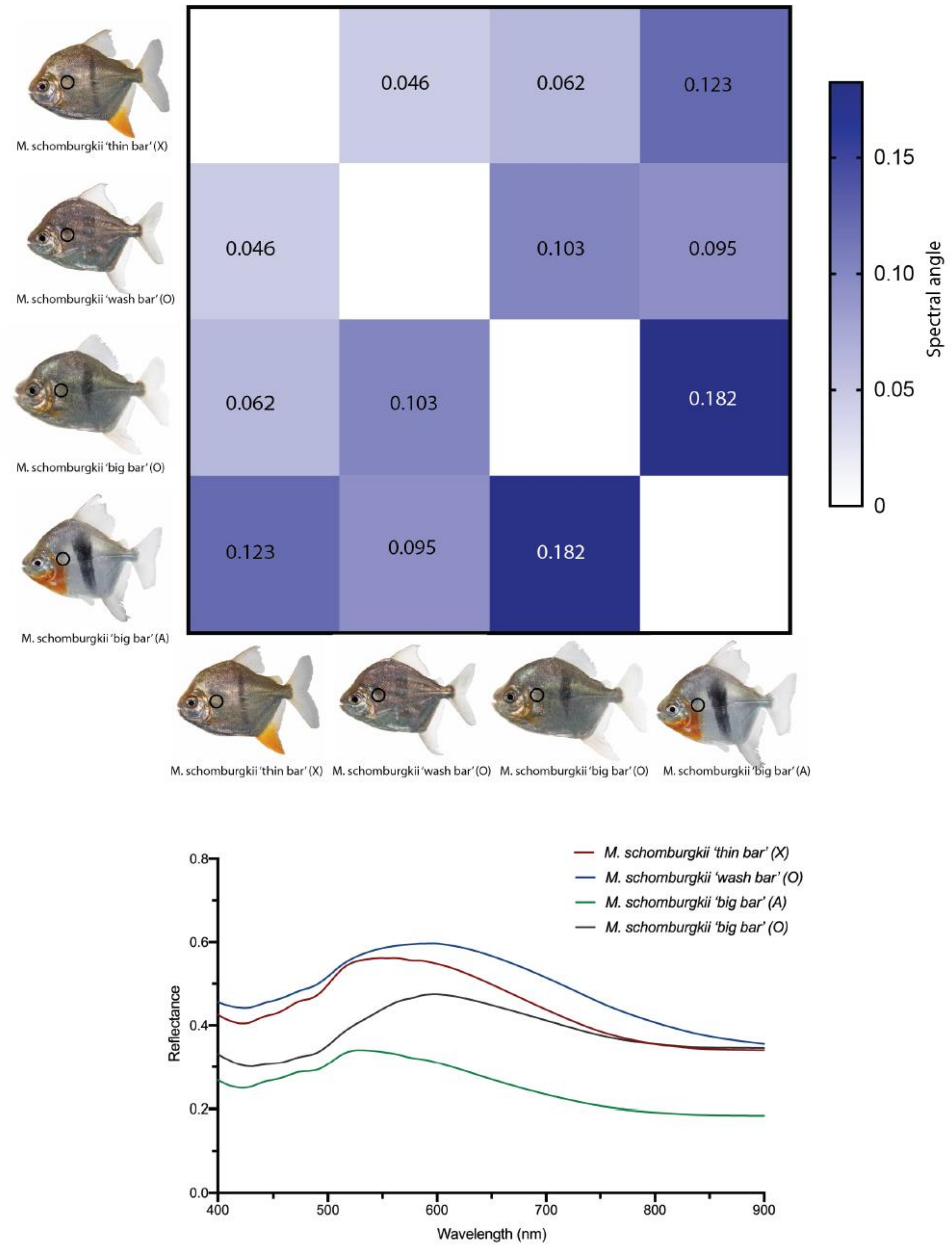

Supplemental Figure S2. A) Spectral angle matrix between the four phenotypic variants of the *Myloplus schomburgkii* species complex. Higher values indicate greater dissimilarity in spectral signatures. B) Mean spectral signatures of the four phenotypical variants of the *M. schomburgkii* species complex. The circles indicate the location of the spectral signature measurement. X = Xingu, A = Araguaia, O = Orinoco.

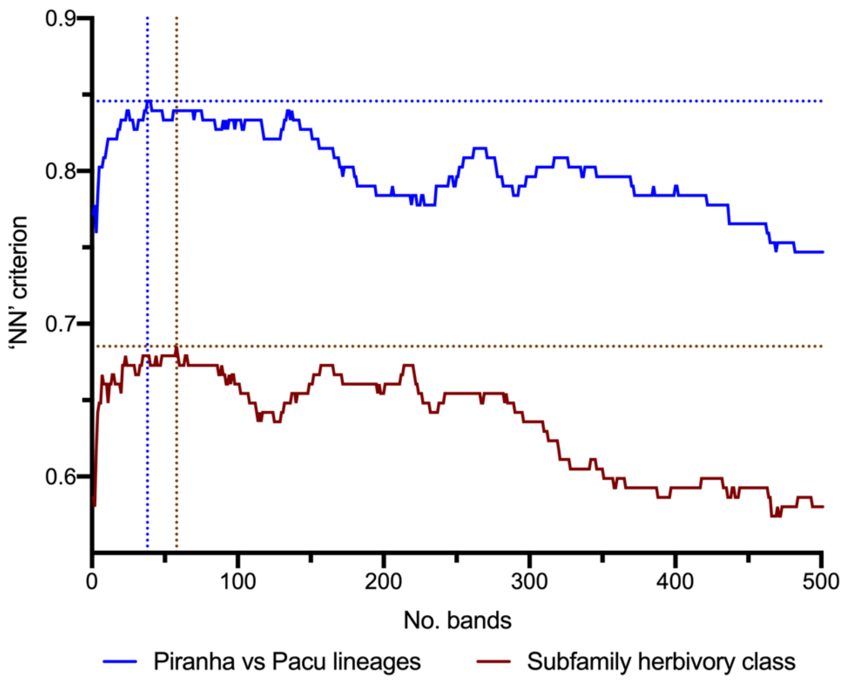

Supplemental Figure S3. Impact of the number of bands on the separability of the spectral signatures of piranha and pacu lineages (blue) and between recognized subfamilies (Colossomatinae = *Colossoma, Piaractus, Mylossoma;* Myleinae = e.g., *Acnodon, Myleus, or Myloplus*; Serrasalminae = e.g., piranhas like *Serrasalmus* or *Pygocentrus* and smaller pacus like *Metynnis* – see Kolmann et al., 2020). Blue dotted lines indicate there is an optimal subset of 38 bands (nearest neighbor (NN) criterion = 0.85) for separating spectral signatures of piranha and pacu lineages. Dotted brown lines indicate there is an optimal subset of 58 bands (NN criterion = 0.67) between subfamilies.

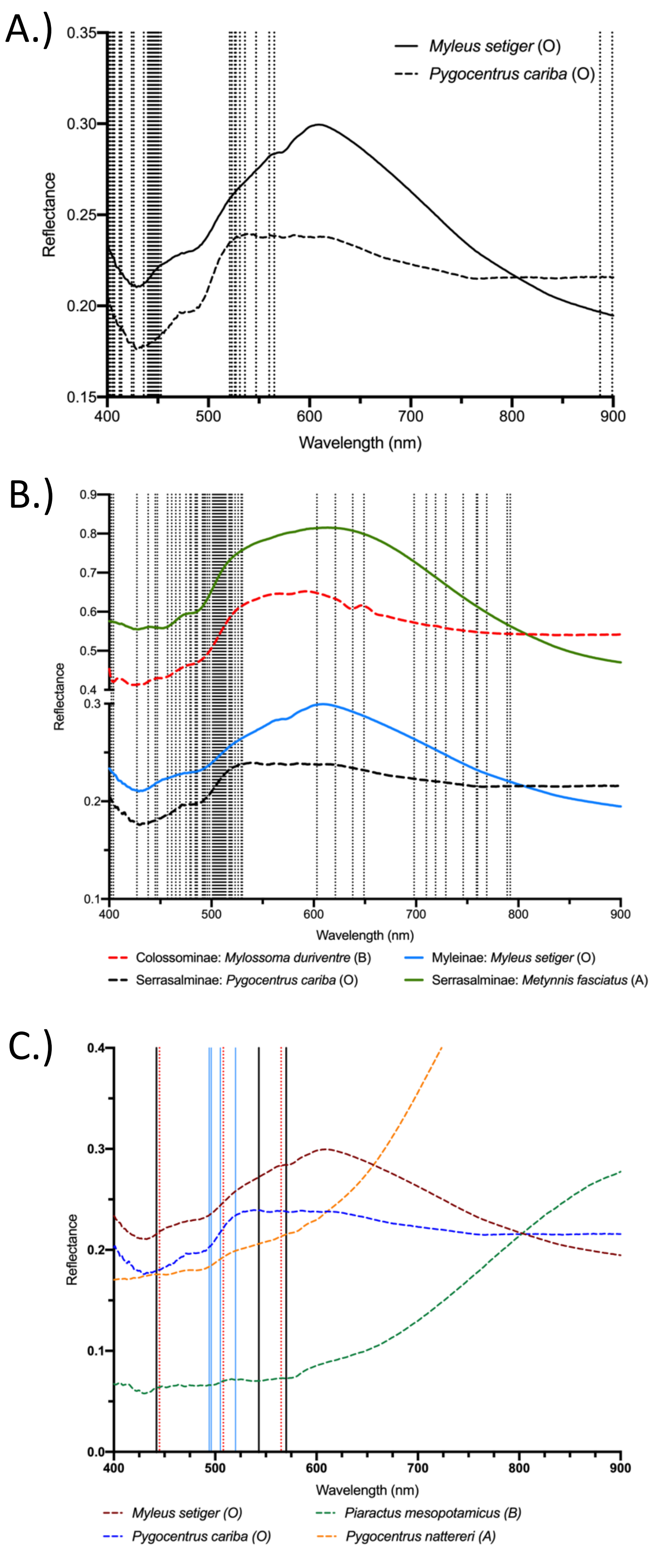

Supplemental Figure S4. Optimal bands (black dotted lines) for separating spectral signatures of A) piranha and pacu lineages; B) subfamilies (Colossomatinae, Myleinae, & Serrasalminae). The spectral signatures of the specimens with the smallest spectral angles to the mean of their respective classes are shown. C) The spectral signatures of the specimens with the smallest (*M. setiger* and *P. cariba*) and largest (*P. mesopotamicus* and *P. nattereri*) spectral angles to the mean piranha and pacu lineage spectral signatures are shown. The solid black vertical lines represent the sensitivity (λmax) of cones for human color vision. The solid black line at the shortest and longest wavelength also approximates the λmax of the river otter's dichromate vision. The dotted red lines represent λmax for bird tetrachromate vision (UV not shown). The solid blue lines represent λmax in fish vision based on levels of chromacy: monochromacy, dichromacy, trichromacy, tetrachromacy, pentachromacy. The sensitivity of rods in freshwater fish vision is also shown.

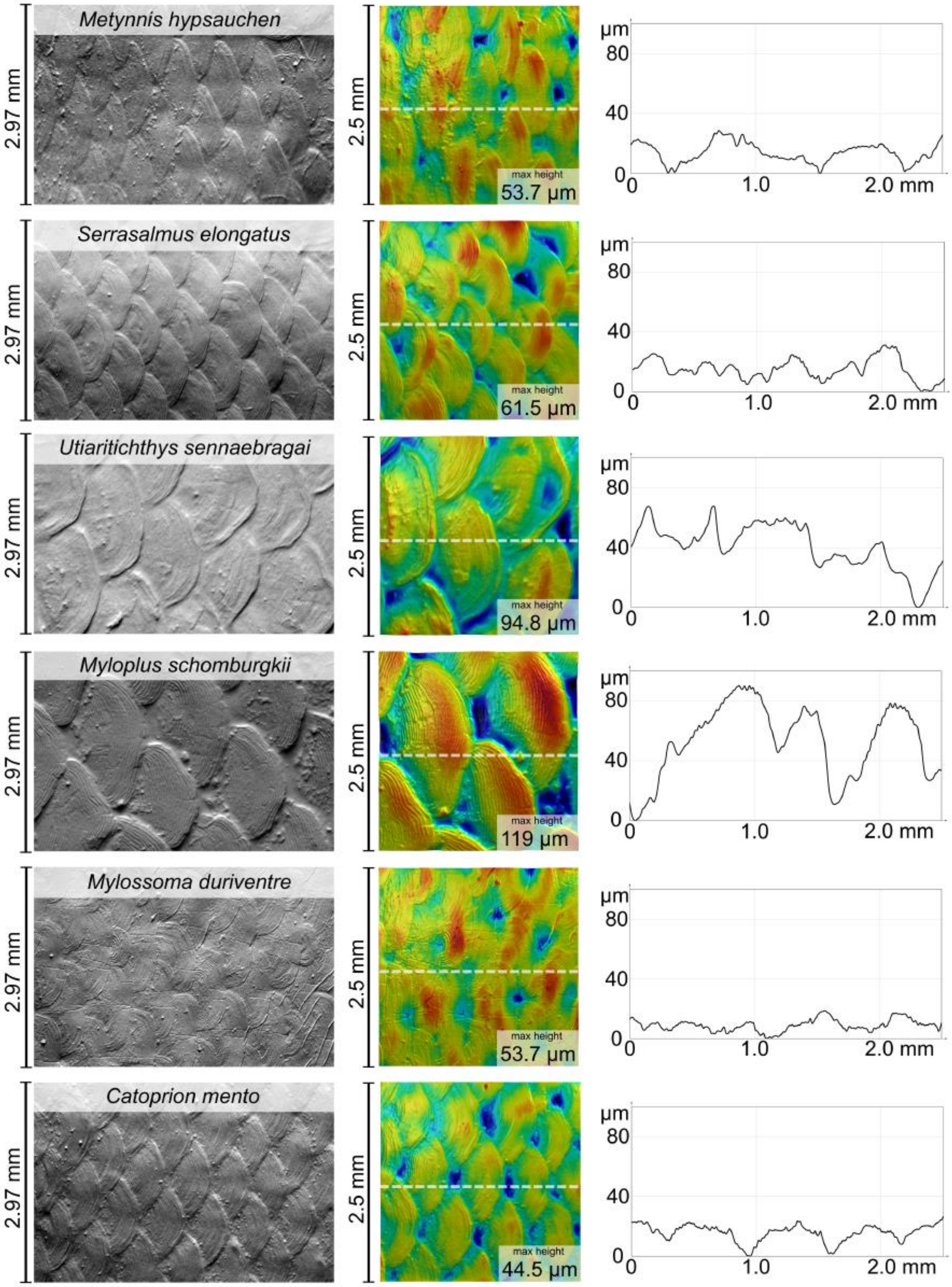

Supplemental Figure S5. Gross scale morphology of six specie of serrasalmids. Left column represents the GelSight surfaces. Centre column represents the topographic map of the scales with red indicating high points, and blue the low points. Right column illustrates a topographic cross section (location of the white dashed line).

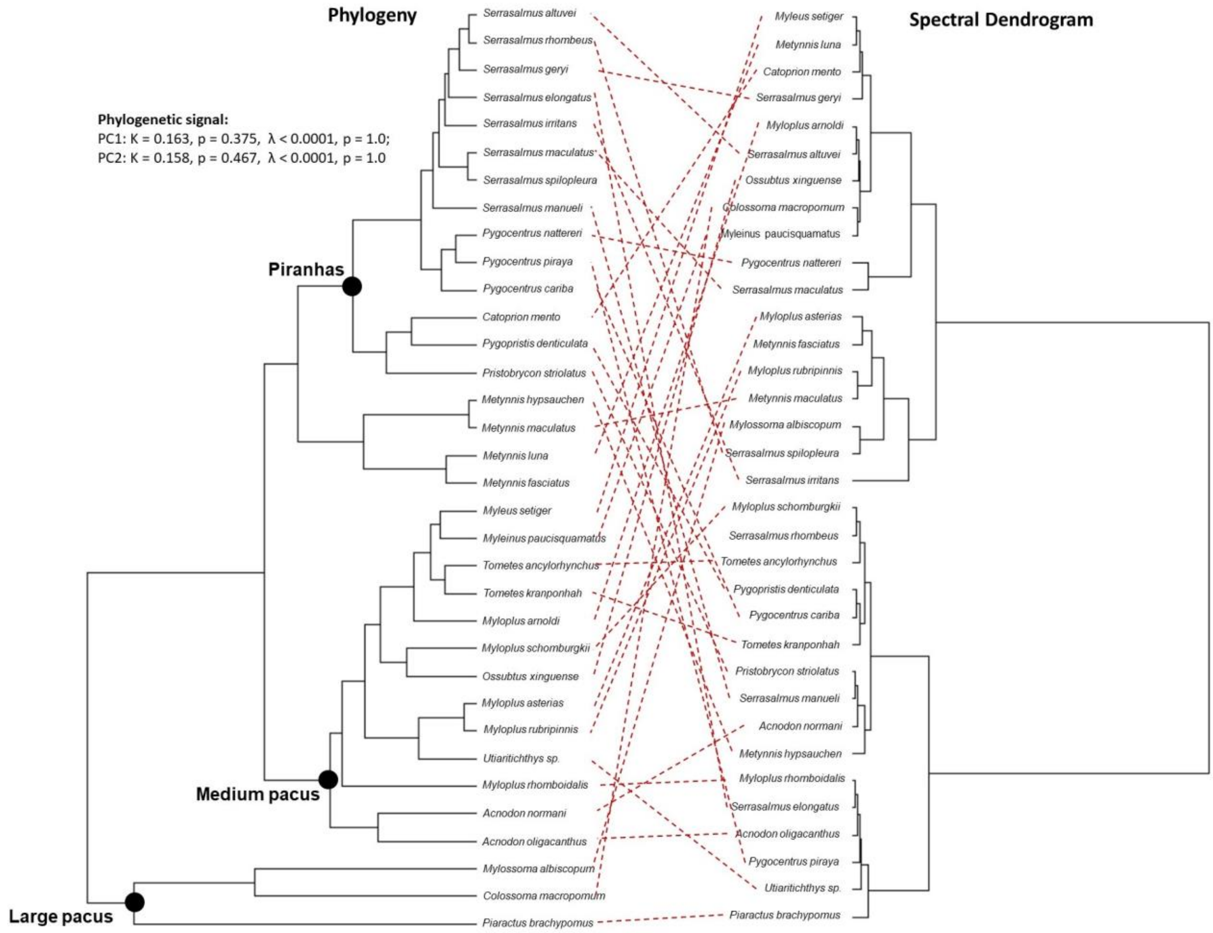

Supplemental Figure S6. spectral signature diversity relative to phylogenetic relatedness in the Serrasalmidae. Phylogeny on left, distance dendrogram of the spectral signatures based on the first two principal components on the right. Dotted lines represent matches between taxa.

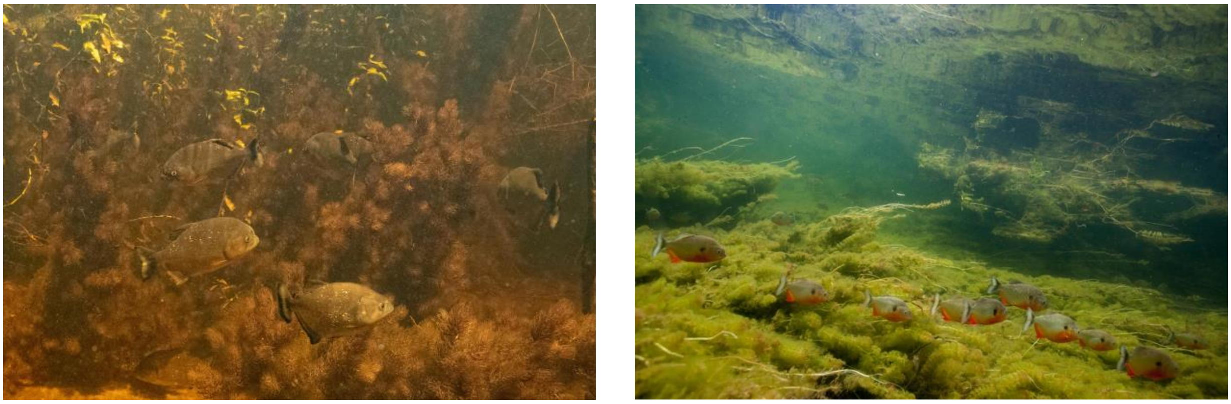

Supplemental Figure S7. Example of two contrasting natural habitats with varying illumination and visibility conditions in which serrasalmids are prevalent. a) Salobra river, Brazil. *Pygocentrus nattereri* and *Serrasalmus maculatus*. b) Orinoco floodplain, Venezuela. School of *Pygocentrus cariba*. Photographs by O. Lucanus
