# Supplementary material for: Hyperspectral data as a biodiversity screening tool can differentiate among diverse Neotropical fishes
Source: Sci Rep. 2021 Aug 9;11:16157. doi: 10.1038/s41598-021-95713-0 (PMC8352966; doi:10.1038/s41598-021-95713-0)
Supplement: Supplementary file 2 — Supplementary Information 2. [file 41598_2021_95713_MOESM2_ESM.docx]

**Hyperspectral data as a biodiversity screening tool can differentiate among diverse Neotropical fishes**

**Kolmann, M.A.^1*,2^, Kalacska M.^3^, Lucanus O.^4^, Sousa L. ^5^, Wainwright D. ^6^, Arroyo-Mora J.P.^7^, Andrade M.C.^8^**

**Affiliations:**

^1^University of Michigan, 1105 N University Ave, Ann Arbor, MI 48109

^2^Royal Ontario Museum, 100 Queens Park, Toronto, ON M5S 2C6

^3^Applied Remote Sensing Lab, McGill University, Montreal, QC H3A 0B9, Canada

^4^Below Water Inc., Vaudreuil-Dorion, QC J7V 0K4, Canada

^5^Laboratório de Ictiologia de Altamira, Universidade Federal do Pará, Altamira, PA, Brazil

^6^Yale University (Peabody Museum)

^7^National Research Council Canada, Ottawa ON

^8^ Núcleo de Ecologia Aquática e Pesca da Amazônia, Universidade Federal do Pará, Belem, PA, Brazil

*corresponding author’s address:

University of Michigan, Museum of Paleontology, 1105 N University Ave, Ann Arbor, MI 48109

Email: mkolmann@gmail.com

Running headline: *spectral signatures for cataloging biodiversity*

**TABLES**

Table S1. List of species included in the study. Each row represents a sample.

| **Genus** | **Species** | **Origin** | **Single/Multi** | **Pacu/Piranha** | **Subfamily** | **Notes** |
| --- | --- | --- | --- | --- | --- | --- |
| *Acnodon* | *normani* | Xingu | M | Md Pacu | Myleinae |  |
| *Acnodon* | *oligacanthus* | Suriname | S | Md Pacu | Myleinae |  |
| *Colossoma* | *macropomum* | Amazon lowlands | M | Lg Pacu | Colossomatinae |  |
| *Metynnis* | *fasciatus* | Araguaia | M | Sm Pacu | Serrasalminae |  |
| *Metynnis* | *hypsauchen* | Suriname | M | Sm Pacu | Serrasalminae |  |
| *Metynnis* | *luna* | Tocantins | M | Sm Pacu | Serrasalminae |  |
| *Metynnis* | *luna* | Orinoco | M | Sm Pacu | Serrasalminae |  |
| *Metynnis* | *maculatus* | Pantanal | S | Sm Pacu | Serrasalminae |  |
| *Metynnis* | *maculatus* | Tocantins | M | Sm Pacu | Serrasalminae |  |
| *Metynnis* | *mola* | Pantanal | S | Sm Pacu | Serrasalminae |  |
| *Mylesinus* | *paraschomburgkii* | Xingu | S | Md Pacu | Myleinae |  |
| *Mylesinus* | *paucisquamatus* | Araguaia | S | Md Pacu | Myleinae |  |
| *Mylesinus* | *paucisquamatus* | Tocantins | S | Md Pacu | Myleinae |  |
| *Myleus* | *setiger* | Orinoco | M | Md Pacu | Myleinae |  |
| *Myleus* | *arnoldi* | Araguaia | M | Md Pacu | Myleinae |  |
| *Myleus* | *arnoldi* | Tocantins | M | Md Pacu | Myleinae |  |
| *Myloplus* | *asterias* | Orinoco | M | Md Pacu | Myleinae |  |
| *Myloplus* | *asterias* ‘yellow’ | Araguaia | M | Md Pacu | Myleinae | Adults and sub adults |
| *Myloplus* | *asterias ‘*Tapajos’ | Tapajos | M | Md Pacu | Myleinae | Adults and sub adults |
| *Myloplus* | *levis* | Araguaia | M | Md Pacu | Myleinae |  |
| *Myloplus* | *levis* | Pantanal | S | Md Pacu | Myleinae |  |
| *Myloplus* | *rhomboidalis* | Xingu | S | Md Pacu | Myleinae |  |
| *Myloplus* | *rhomboidalis* | Tocantins | S | Md Pacu | Myleinae |  |
| *Myloplus* | *rubripinnis* | Orinoco | M | Md Pacu | Myleinae |  |
| *Myloplus* | *schomburgkii ‘*big bar’ | Araguaia | M | Md Pacu | Myleinae |  |
| *Myloplus* | *schomburgkii ‘*thin bar’ | Xingu | M | Md Pacu | Myleinae |  |
| *Myloplus* | *schomburgkii ‘*big bar’ | Orinoco | M | Md Pacu | Myleinae |  |
| *Myloplus* | *schomburgkii ‘*wash bar’ | Orinoco | M | Md Pacu | Myleinae |  |
| *Myloplus* | *torquatus* | Orinoco | M | Md Pacu | Myleinae | Adults and sub adults |
| *Mylossoma* | *duriventre* | Pantanal | S | Lg Pacu | Colossomatinae |  |
| *Ossubtus* | *xinguensis* | Xingu | M | Md Pacu | Myleinae |  |
| *Piaractus* | *mesopotamicus* | Pantanal | S | Lg Pacu | Colossomatinae |  |
| *Pristobrycon* | *striolatus* | Amazon lowlands | S | Piranha | Serrasalminae |  |
| *Pygocentrus* | *cariba* | Orinoco | M | Piranha | Serrasalminae |  |
| *Pygocentrus* | *nattereri* | Araguaia | S | Piranha | Serrasalminae |  |
| *Pygocentrus* | *nattereri* | Amazon lowlands | M | Piranha | Serrasalminae |  |
| *Pygocentrus* | *nattereri* | Pantanal | S | Piranha | Serrasalminae |  |
| *Pygocentrus* | *piraya* | Tocantins | S | Piranha | Serrasalminae |  |
| *Pygopristis* | *denticulata* | Suriname | S | Piranha | Serrasalminae |  |
| *Catoprion* | *mento* | Orinoco | S | Piranha | Serrasalminae |  |
| *Serrasalmus* | *elongatus* | Amazon lowlands | M | Piranha | Serrsalminae | Adult and sub adult |
| *Serrasalmus* | *geryi* | Araguaia | M | Piranha | Serrasalminae | Adult and sub adult |
| *Serrasalmus* | *hollandi* | Amazon lowlands | S | Piranha | Serrasalminae |  |
| *Serrasalmus* | *humeralis* | Araguaia | M | Piranha | Serrasalminae | Adult and sub adult |
| *Serrasalmus* | *irritans* | Orinoco | S | Piranha | Serrasalminae |  |
| *Serrasalmus* | *maculatus* | Araguaia | S | Piranha | Serrasalminae |  |
| *Serrasalmus* | *maculatus* | Amazon lowlands | M | Piranha | Serrasalminae |  |
| *Serrasalmus* | *maculatus* | Pantanal | S | Piranha | Serrasalminae |  |
| *Serrasalmus* | *manueli* | Xingu | S | Piranha | Serrasalminae |  |
| *Serrasalmus* | *marginatus* | Pantanal | S | Piranha | Serrasalminae |  |
| *Serrasalmus* | *odyssei* | Amazon lowlands | S | Piranha | Serrasalminae |  |
| *Serrasalmus* | *rhombeus* | Amazon lowlands | M | Piranha | Serrasalminae | Adult and sub adult |
| *Serrasalmus* | *rhombeus* | Araguaia | M | Piranha | Serrasalminae |  |
| *Serrasalmus* | *sanchezi* | Amazon lowlands | M | Piranha | Serrasalminae |  |
| *Serrasalmus* | *serrulatus* | Amazon lowlands | S | Piranha | Serrasalminae |  |
| *Serrasalmus* | *spilopleura* | Tocantins | M | Piranha | Serrasalminae |  |
| *Serrasalmus* | sp. ‘Llanos’ | Orinoco | S | Piranha | Serrasalminae |  |
| *Tometes* | *ancylorhynchus* | Araguaia | M | Md Pacu | Myleinae |  |
| *Tometes* | *ancylorhynchus* | Xingu | M | Md Pacu | Myleinae |  |
| *Tometes* | *kranponhah* | Xingu | S | Md Pacu | Myleinae |  |
| *Tometes* | *siderocarajensis* | Tocantins | M | Md Pacu | Myleinae |  |
| *Tometes* | sp. ‘Tapajos’ | Tapajos | M | Md Pacu | Myleinae | Adults and juveniles |
| *Utiaritichthys* | *sennaebragai* | Tapajos | S | Md Pacu | Myleinae |  |
| *Utiaritichthys* | *cf. longidorsalis* | Xingu | S | Md Pacu | Myleinae |  |

Table S2. Species sampled with gel-based profilometry.

| **Species** | **n** | **Harvard Museum of Comparative Zoology (MCZ) #** |
| --- | --- | --- |
| *Catoprion mento* | 4 | 48487, 19087 (2), 19079 |
| *Metynnis hypsauchen* | 3 | 30126, 92831 (2) |
| *Myloplus schomburgkii* | 5 | 19307 (5) |
| *Mylossoma duriventre* | 3 | 92830, 92828, 172763 |
| *Serrasalmus elongatus* | 9 | 20592 (3), 19009, 19176, 19163, 54177 (3) |
| *Utiaritichthys sennaebragai* | 3 | 19026, 19027, 19028 |
